# Advances in the Release of Amide‐Containing Molecules

**DOI:** 10.1002/chem.202404413

**Published:** 2025-01-28

**Authors:** Thomas Wharton, David R. Spring

**Affiliations:** ^1^ Yusuf Hamied Department of Chemistry University of Cambridge Lensfield Road Cambridge, CB2 1EW UK

**Keywords:** Amides, Linezolid, Prodrugs, Release, Self-immolative

## Abstract

The ability to release a molecule from a larger construct in a controlled manner is of great importance to produce effective prodrugs, antibody‐drug conjugates, and chemical probes. Amides are ubiquitous functional groups and yet methods to utilise them as molecular release handles are seldom reported. This concept article highlights the advances made in amide release strategies and how these approaches have been utilised.

## Graphical abstract

The ability to release a molecule from a larger construct in a controlled manner is of great importance to produce effective prodrugs, antibody‐drug conjugates, and chemical probes. Amides are ubiquitous functional groups, yet methods to utilise them as molecular release handles are seldom reported. This concept highlights advances made in amide release strategies and how these approaches have been utilised.

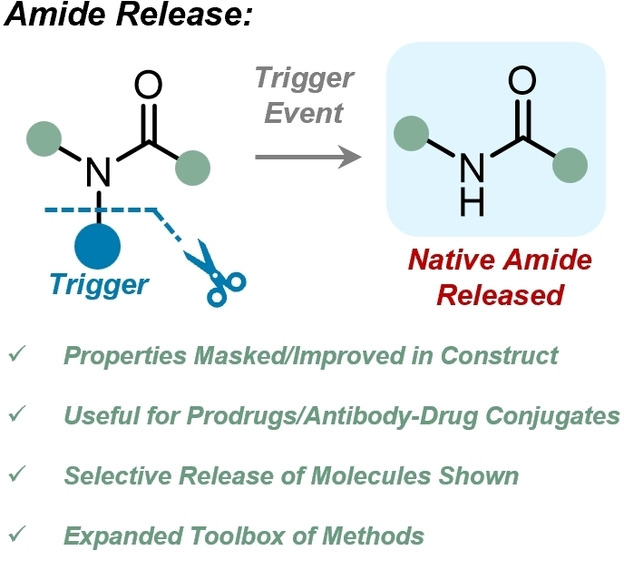

## Introduction

Strategies that allow the selective release of a payload in its native form from a larger construct have found utility in many areas of chemical and biological science, such as in prodrugs and chemical probes,[^1^, ^2^, ^3^, ^4^, ^5^, ^6^, ^7^, ^8^, ^9^] antibody‐drug conjugates (ADCs),[^10^, ^11^, ^12^, ^13^, ^14^, ^15^, ^16^] materials science[^17^, ^18^, ^19^, ^20^, ^21^] and solid supported chemical synthesis.^[22]^

**Scheme 1:**
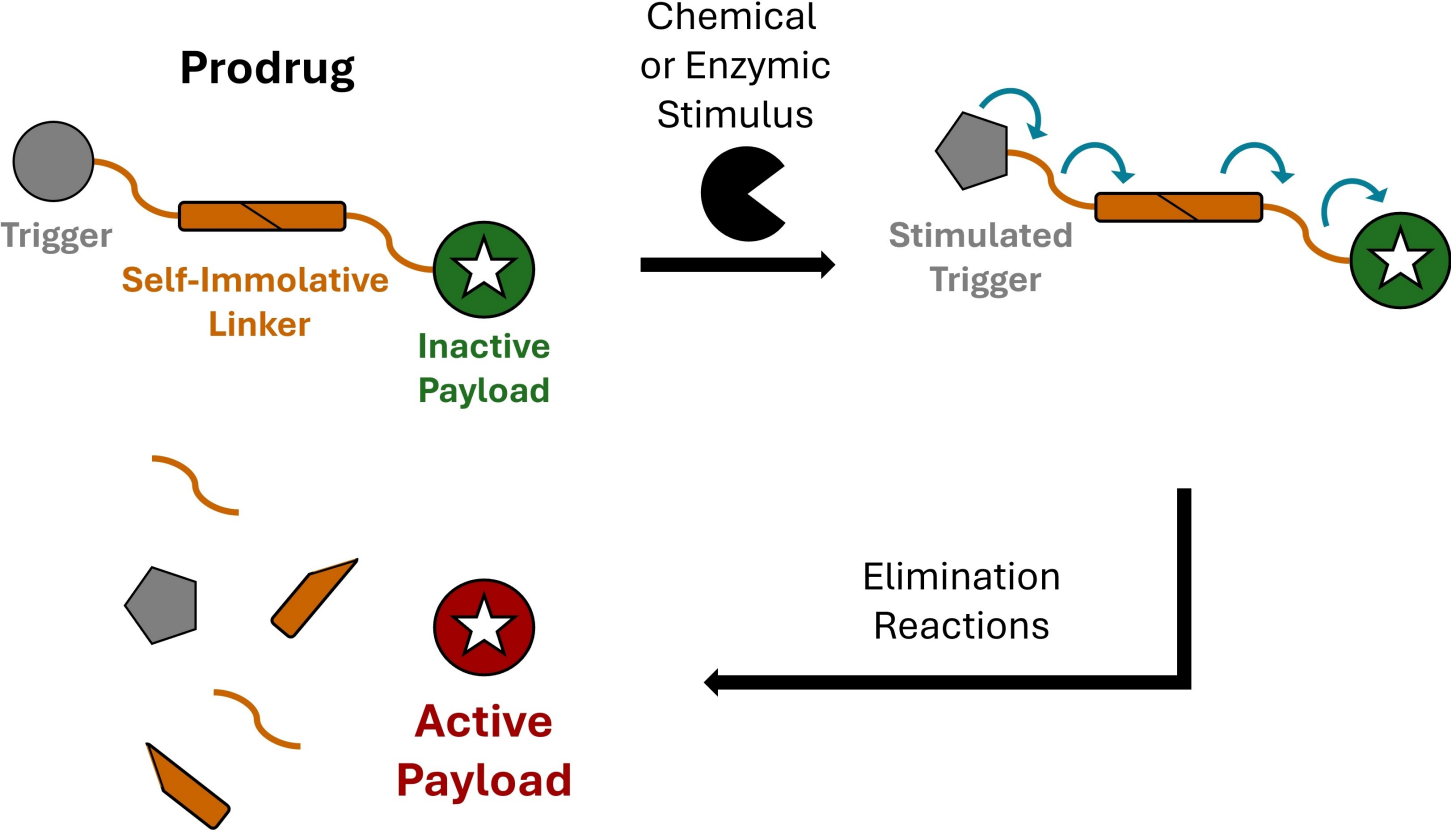
Schematic of a self‐immolative linker system, showing how a stimulus causes fragmentation via spontaneous elimination reactions.

A major family of selective release linkers are the self‐immolative or ‘traceless’ linker systems that allow the fragmentation of a construct upon a specific trigger event, releasing the molecule(s) of interest (Scheme 1).[^23^, ^24^] Ideally, this allows for an initial masking of certain properties of the molecule while included in the construct, after which these properties are restored upon a controlled release. These approaches also enable existing molecules of interest to be used in novel constructs, expediting research programs and allowing their exploitation.

Many self‐immolative linker systems utilise free amine,[^25^, ^26^, ^27^] hydroxy,[^9^, ^12^, ^15^, ^16^] or thiol^[14]^ groups on the payload as the linkage point due to their prevalence, ease of synthetic manipulation, and the multitude of different release methodologies described.^[24]^ Indeed, these three key functional handles are found in all FDA‐approved ADCs with cleavable linkers.^[10]^ However, evidently not all molecules possess amines, alcohols, and thiols, and whilst often these groups can be incorporated into the structure, this inherently affects the properties and function of the released molecule and so any changes must be carefully planned. The ability to release a molecule of interest in its native form using other functional groups, therefore, is highly attractive.

Amides are a ubiquitous functional group yet methods for their use as self‐immolative linker handles have been seldom reported. This concept article discusses methods developed to release amide‐containing molecules, highlighting their synthesis, release mechanism, and utility. Undoubtedly, the largest interest in the controlled release of molecules is in the generation of novel prodrugs and ADCs for targeted therapies and indeed all reported amide release methods have focused on the generation of therapeutics. Like all self‐immolative systems, amide release methods lead to the formation of by‐products whose biological effects must be considered before a method is utilised in therapeutic generation. Nevertheless, as a main aim of drug release is generating targeted therapies, any possible by‐product toxicity may be outweighed by the benefit of reduced off‐target toxicity of the parent drug, as is the case in the self‐immolative FDA‐approved ADCs. As the reported amide‐release methods utilise bespoke trigger/linker systems the toxicity of fragments is unknown in many cases due to the early stage development of the systems. To this end, further studies are required in each case.

Though they are beyond the scope of this concept article, the authors note that methods have also been described to release other underrepresented functional groups such as imines,^[28]^ pyrimidones,^[29]^ imides,^[30]^ thioamides,^[31]^ and ketones.^[32]^

### Key Advances

**Scheme 2:**
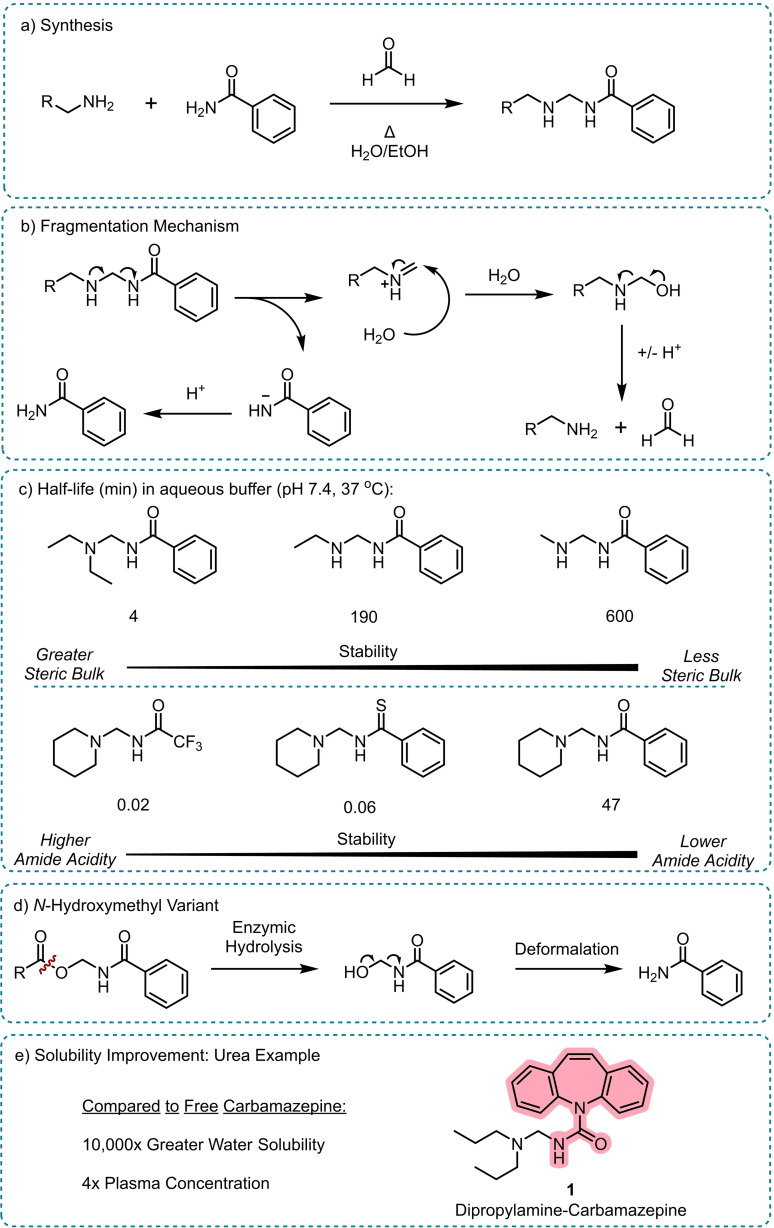
Amide release from *N*‐Mannich base prodrugs.^[33]^ a) Synthesis, b) Proposed fragmentation mechanism in aqueous media, c) Effect of steric hinderance and parent amide pKa on prodrug half‐life, d) *N*‐Hydroxymethyl variant based on ester hydrolysis,^[34]^ e) Dipropylamine prodrug of carbamazepine, **1**, showed greatly enhanced solubility compared to the free drug.^[35]^ The released drug is highlighted.

The first reported systems for amide release focused on capping the amide moiety to mask its properties, with the aim of producing prodrugs to enhance solubility and thus aid administration of the parent molecule. Bundgaard *et. al*.^[33]^ described the stability and reactivity of prodrugs of amide, sulphonamide, and urea containing molecules masked as *N*‐Mannich bases (*N*‐aminomethyl derivatives) (Scheme 2). The prodrugs were synthesised from parent model amides via Mannich reactions of formaldehyde and a variety of amines with varied steric and electronic properties. The prodrugs were then incubated in aqueous buffers at 37 °C to determine their stability. In all cases the *N*‐Mannich bases decomposed to release the parent amide and formaldehyde, presumably via a retro‐Mannich mechanism (Scheme 2b), with half‐lives ranging from seconds to 26 hours.

An increased rate of decomposition correlated with an increased acidity of the parent amide or increased steric bulk on the amine component (Scheme 2c). It was postulated that steric bulk around the amine reduced the solvation of the nitrogen lone pair, thus increasing its electron‐donating power to trigger the decomposition. An analogous strategy was also reported utilising an *N*‐hydroxymethyl instead of *N*‐aminomethyl mask where the labile linkage was revealed only by ester hydrolysis, improving the selectively of release (Scheme 2d).^[34]^

In a further study, it was reported that **1**, a dipropylamine prodrug of the urea containing epilepsy drug carbamazepine, had a 10^4^‐fold greater solubility in water than native carbamazepine; this in turn led to a 4‐fold higher plasma concentration when administered intramuscularly (Scheme 2e).^[35]^ Once in the blood stream, the prodrug would decompose to release the free urea drug. This shows that the amine capping group can alter the properties compared to the parent drug, in this case increasing solubility to improve the administration of the therapy.

**Scheme 3:**
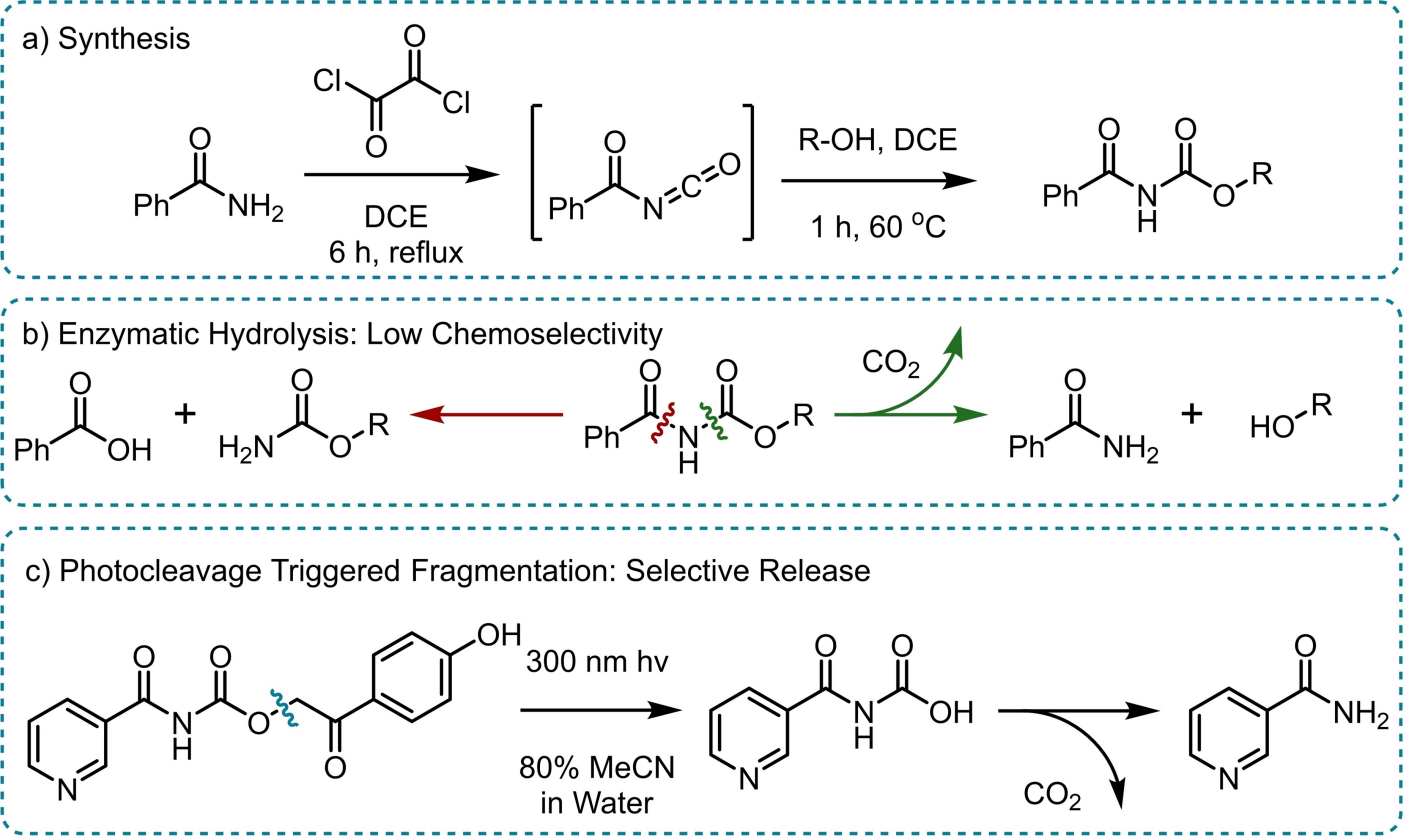
Amide release from *N*‐alkyloxycarbonyl prodrugs. a) Synthesis,^[36]^ b) Enzymic hydrolysis in plasma leads to competing C−N bond fission pathways, c) Cleavage of a photolabile C−O bond allows for selective release of nicotinamide after decarboxylation.^[38]^

Bundgaard and co‐workers also explored the use of *N*‐acylation and *N*‐alkyloxycarbonylation as amide masks, with the release mechanism relying on hydrolysis by undisclosed enzymes found in plasma upon the prodrug reaching the blood stream (Scheme 3).^[36]^ Whilst the acyl‐masked amides proved far more stable than the *N*‐Mannich bases in aqueous buffer, the selectively to which C−N bond is cleaved in plasma was found to be difficult to control, leading to unwanted carboxylic acid by‐products thus precluding this methods greater utility (Scheme 3b).

To avoid the unselective nature of enzymic C−N bond fission in *N*‐acyl carbamates, Helquist and co‐workers utilised chromophores to introduce selectivity to the amide release via photocleavage (Scheme 3c).[^37^, ^38^] Initial studies using coumarin as a chromophore allowed for the successful release of nicotinamide upon irradiation with 300 nm UV, however, the authors noted a slow rate of release and a quantum yield of just 1 %.

By switching to a *p*‐hydroxyphenacyl‐based chromophore the quantum yield was increased up to 11 %. However, the requirement of an 80 % MeCN buffer system and UV irradiation greatly precludes the methods use in a biological setting. Nevertheless, the photocaged nicotinamides were found to be stable to hydrolysis over 24 hours in the absence of light.

**Scheme 4:**
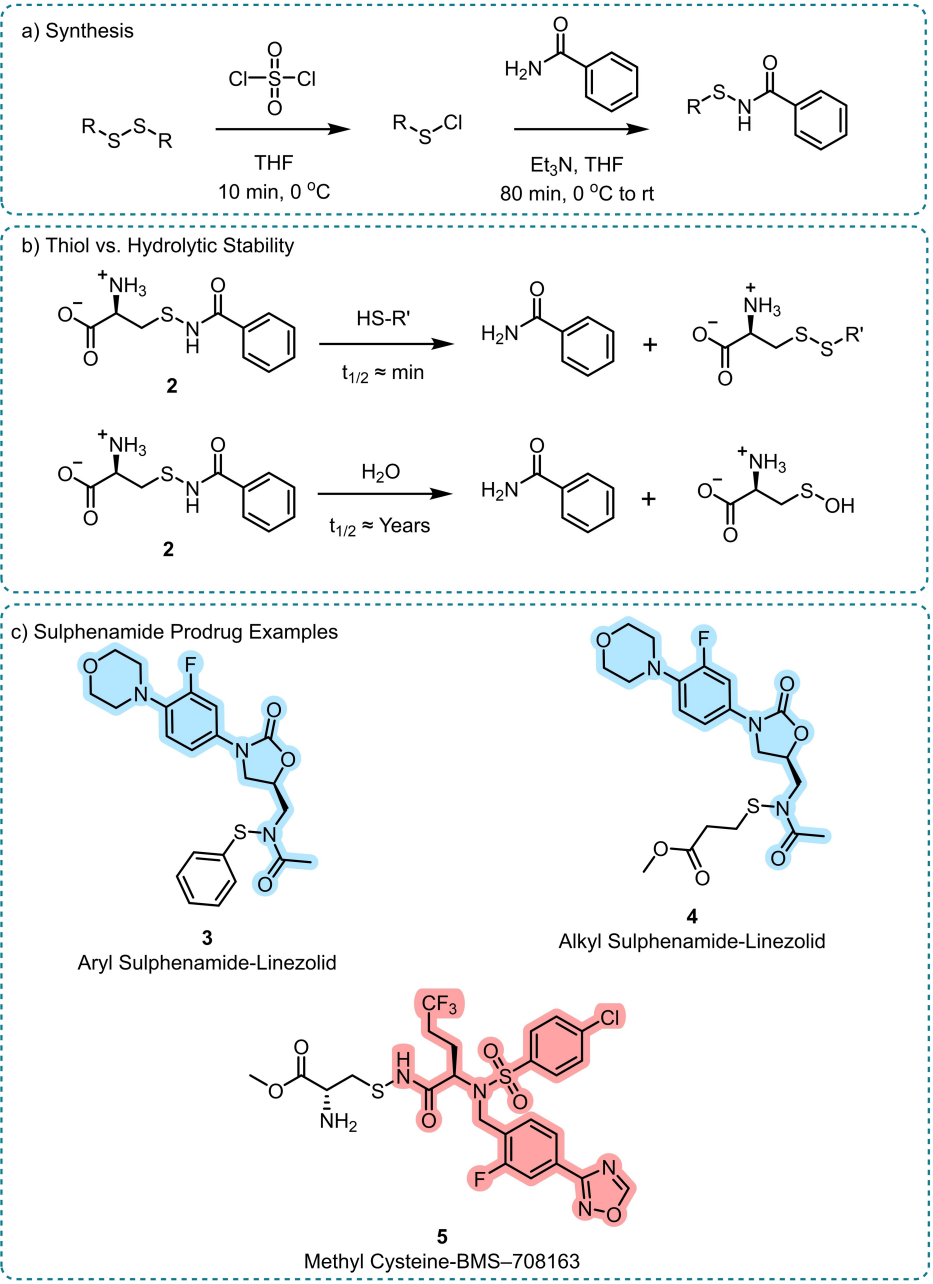
Amide release from sulphenamide prodrugs.[^39^, ^41^] a) Synthesis, b) Stability with and without the presence of thiols in aqueous media, c) Example sulphenamide prodrugs. Linezolid prodrugs **3** and **4** showed excellent fragmentation rates in the presence of thiol. Prodrug **5** exhibited improved solubility and drug plasma concentration compared to the parent Alzheimer's drug candidate BMS–708163. The released drug is highlighted in each case.

Stella and co‐workers reported a hydrolytically stable sulphenamide‐based amide prodrug approach with the aim to increase the solubility and membrane penetration properties compared to the parent amide drugs (Scheme 4).^[39]^ The method relies on the polarisation of the N−S bond and the subsequent electrophilicity of the sulphur, allowing its displacement by free thiols in the blood stream. Via substitution on the sulphenamide, the method aimed to allow the hydrophilicity or lipophilicity to be adapted according to the need of the parent amide to improve solubility or cellular uptake. As with the *N*‐Mannich base prodrug approach, the conversion of a secondary amide to a prodrug also removed the hydrogen bonding capabilities which further affected compound solubility.

The sulphenamides proved to be vastly more hydrolytically stable than the *N*‐Mannich bases, with a cystine‐benzamide example (**2**) having an extrapolated half‐life of over six years at pH 6 (Scheme 4b).^[39]^ Once excess cysteine was added as a source of free thiol, the rate of fragmentation increased by four‐orders of magnitude, showing the viability of the method to release the native amide only in the presence of thiol. Furthermore, linezolid‐sulphenamides **3** and **4** were incubated in beagle plasma where full release of native linezolid was observed within two minutes, showcasing the utilisation of both alkyl and aryl sulphenamides (Scheme 4c). In a subsequent study, these linezolid prodrugs were tested with the hypothesis that the capped amide and additional lipophilicity of the sulphenamide moiety would improve their cell membrane permeability.^[40]^ Unfortunately, no overall improved permeability was observed, which the authors report as being due to free thiols on the surface of the cell membranes prematurely degrading the sulphenamide moiety.

Improved solubility was exemplified using cystine‐based prodrug **5** of the candidate Alzheimer's drug BMS‐708163 (Scheme 4c; red).^[41]^ **5**, given orally as a dry powder, showed equivalent blood exposure in beagles to a polyethylene glycol (PEG) encapsulated formulation of BMS‐708163, suggesting that the sulphonamide prodrug strategy enabled a large solubility increase without the need for extensive formulation research. In this example, premature prodrug degradation did not hinder the uptake of BMS‐708163, though it is of note that **5** showed excellent pH 1 to 4 stability, suggesting the prodrug could pass through the gastrointestinal tract intact.

**Scheme 5:**
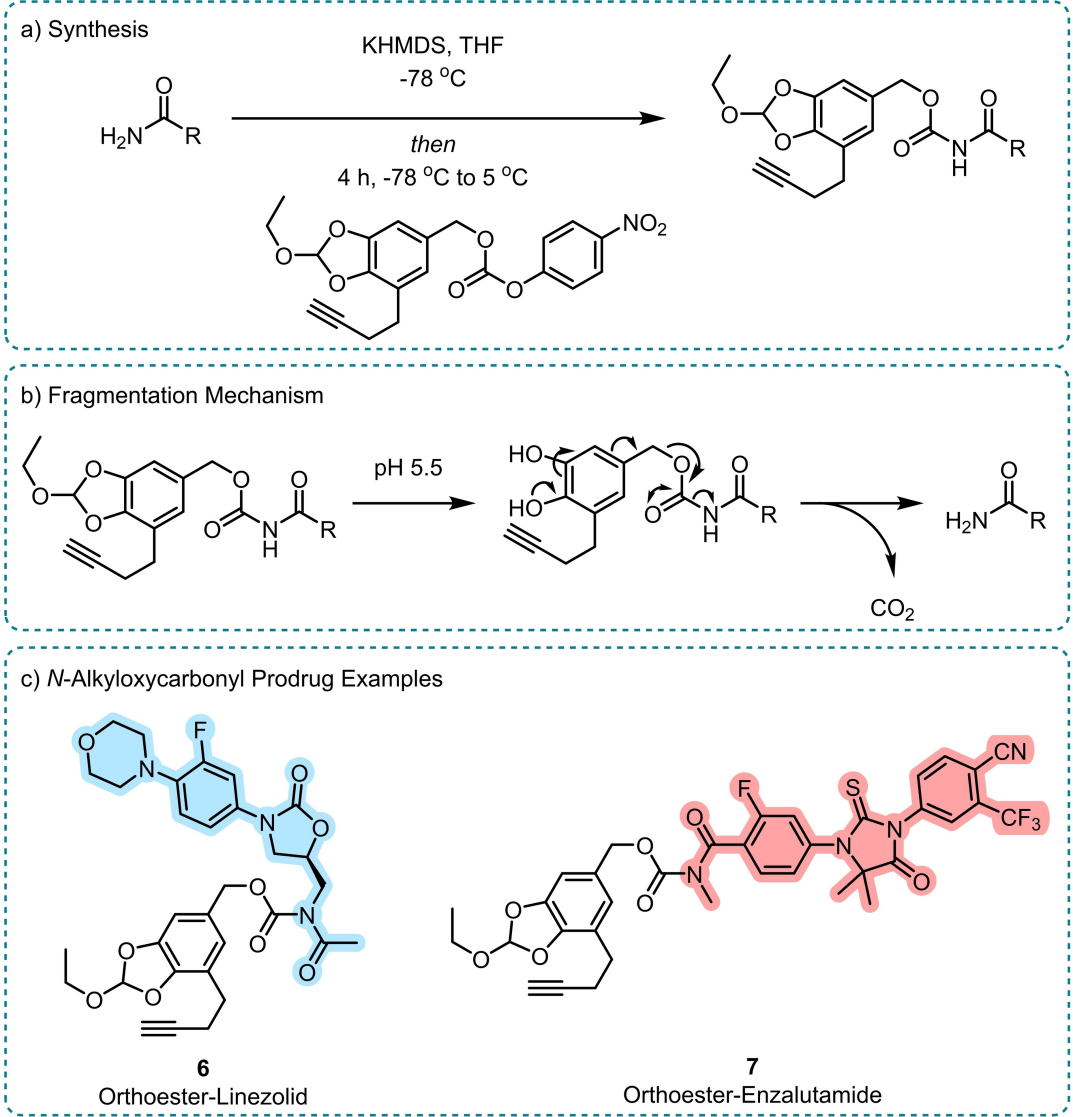
Amide release from acid cleavable *N*‐alkyloxycarbonyl prodrugs.^[42]^ a) Synthesis, b) Fragmentation mechanism triggered by an acid labile orthoester moiety that reveals a phenol poised for 1,6‐benzylic elimination, c) Acid‐labile linezolid **6** and enzalutamide **7** prodrugs. The released drug is highlighted in each case. KHMDS=Potassium bis(trimethylsilyl)amide.

Like Helquist and co‐workers, Petrini *et al*. aimed for amide release via an *N*‐alkyloxycarbonyl group, utilising an orthoester‐based acid cleavable trigger to afford selectivity (Scheme 5).^[42]^ The use of this aryl orthoester group was previously described for the release of amines via carbamates, becoming labile below pH 5.5.^[43]^ Petrini *et al*. combined this trigger with a variety of amides to generate *N*‐alkyloxycarbonyl prodrugs, such as linezolid **6** and enzalutamide **7** (Scheme 5c), which demonstrated up to 80 % amide drug release in 24 hours at pH 5.5 and only minor degradation in plasma and pH 7.4 buffer.^[42]^

**Scheme 6:**
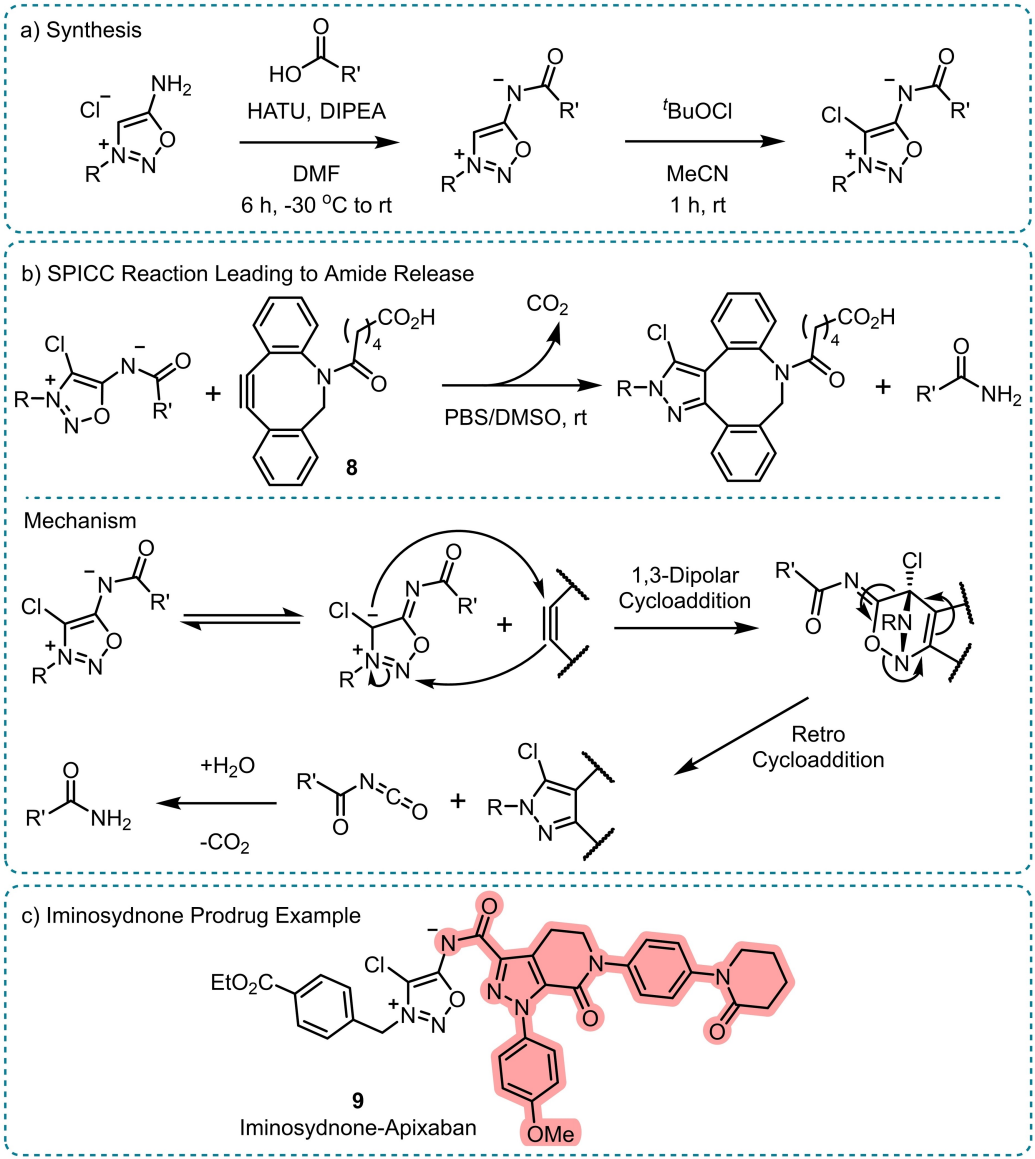
Amide release from iminosydnone prodrugs.^[44]^ a) Synthesis, b) Strain‐promoted iminosydnone–cyclooctyne cycloaddition (SPICC) reaction causing the release of the amide; reaction scheme and mechanism, c) Example iminosydnone‐apixaban prodrug **9**. The released drug is highlighted. HATU=Hexafluorophosphate azabenzotriazole tetramethyl uronium; DIPEA=*N*,*N*‐Diisopropylethylamine; PBS=Phosphate buffered saline.

Feng *et al*. built upon a successful click‐to‐release strategy and adapted it to enable amide release (Scheme 6).^[44]^ Earlier work described the use of strain‐promoted iminosydnone–cyclooctyne cycloaddition (SPICC) to release sulphonamides, carbamates, and ureas upon the click reaction.^[45]^ However, the reaction kinetics to release amide analogues proved too slow to be effective. Computational studies correctly proposed that 4‐chloro‐iminosydnones would undergo SPICC reactions at a much faster rate,^[46]^ enabling a controlled and selective amide release for the first time (Scheme 6b). A 4‐chloro‐iminosydnone prodrug of apixaban, **9** (Scheme 6c), exhibited excellent SPICC reaction kinetics with dibenzocyclooctyne (DBCO) acid **8**, with 90 % free apixaban release detected within 2 hours.^[44]^ The prodrug also showed good aqueous stability with ~10 % degradation over 10 days at room temperature and physiological pH. Using this method, it was envisaged that amide containing drugs could be released *in vivo* from larger constructs by the selective addition of strained alkyne reagents.

**Scheme 7:**
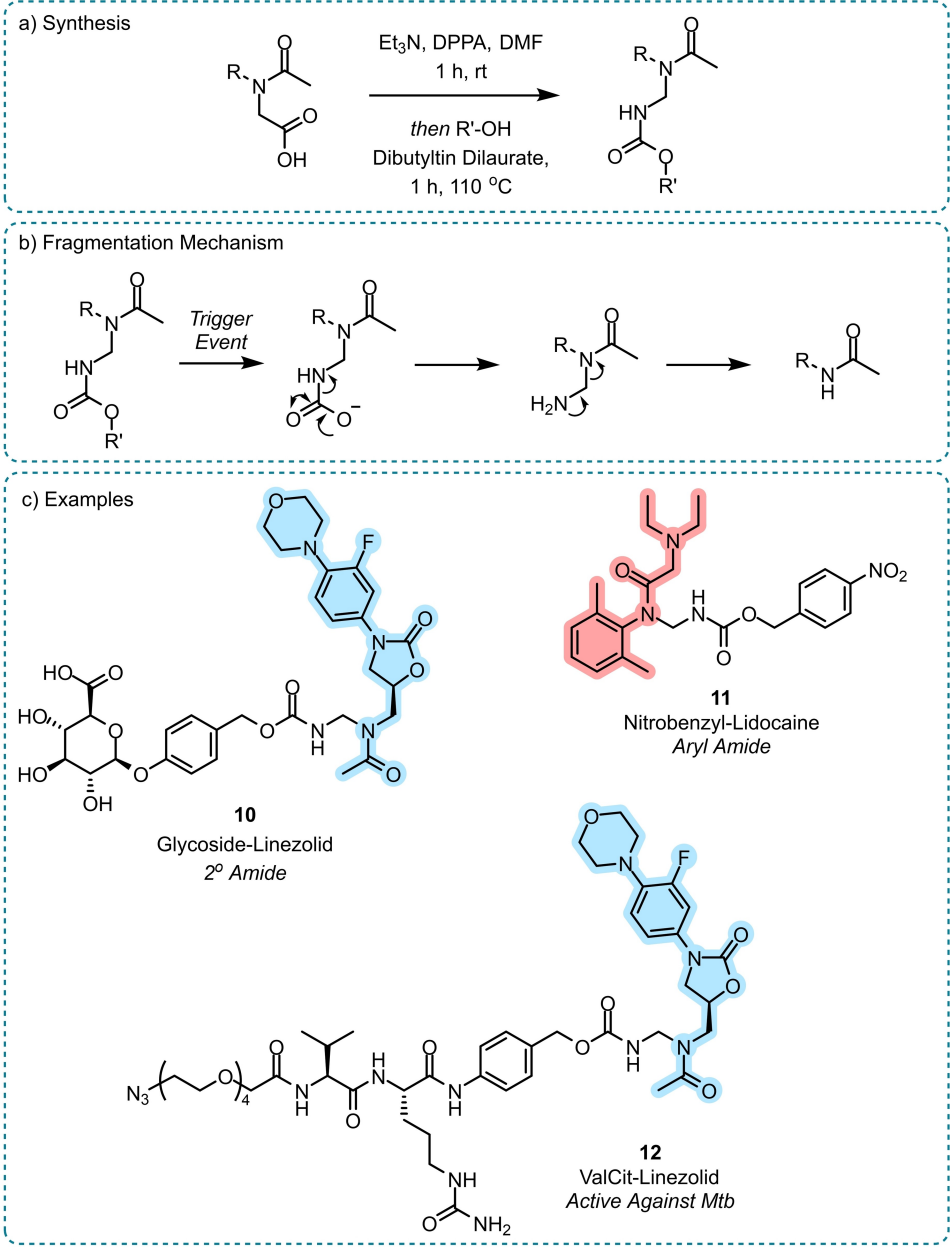
Amide release via aminomethyl carbamate linkages.^[47]^ a) Synthesis, b) Fragmentation mechanism; trigger event leads to classical carbamate degradation to reveal an aminomethyl amide that then undergoes an aminal‐type fragmentation, c) Example amide prodrugs highlighting the range of amide and trigger types tolerated. ValCit‐linezolid **12** showed equipotency to native linezolid against *Mtb* once stimulated to fragment. The released drug is highlighted in each case. DPPA=Diphenylphosphoryl azide.

Wharton *et al*. achieved a general amide release utilising a cleavable carbamate linkage to reveal an *N*‐aminomethyl amide that fragments via aminal‐type degradation (Scheme 7).^[47]^ It was envisaged that a trigger event would cause fragmentation of the construct via 1,6‐benzylic elimination, followed by the release of carbon dioxide to reveal an unstable *N‐*aminomethyl amide that could decompose (akin to that exemplified by Bundgaard and co‐workers) to release the native amide (Scheme 7b). The method was shown to be compatible with a range of amide‐types and triggers releasing primary (levetiracetam), secondary (linezolid), and aryl (lidocaine) amides using common prodrug triggers such as nitrobenzyl, valine‐citrulline (ValCit), and glucuronide motifs (Scheme 7c). Full release of the payloads was achieved within 24 hours regardless of the type of trigger event, with the release kinetics comparable to other aniline‐based self‐immolative fragmentation systems^[26]^ and aminomethyl degradation.^[33]^

The aminomethyl carbamate linkage exhibited stability in human plasma and physiological pH (4.0–9.2) for greater than 10 days for all amide‐release examples.^[47]^ A sulphonamide release was attempted utilising sulfamethizole, however the linkage proved unstable, prematurely releasing the drug in neutral and basic media.

The method was showcased in the release of linezolid (a potent *Mycobacterium tuberculosis* (*Mtb)* antibiotic) from a ValCit‐triggered prodrug, **12**. Studies showed the prodrug selectively released linezolid in the presence of cathepsin B protease and showed equipotency to free linezolid against *Mtb*.

**Scheme 8:**
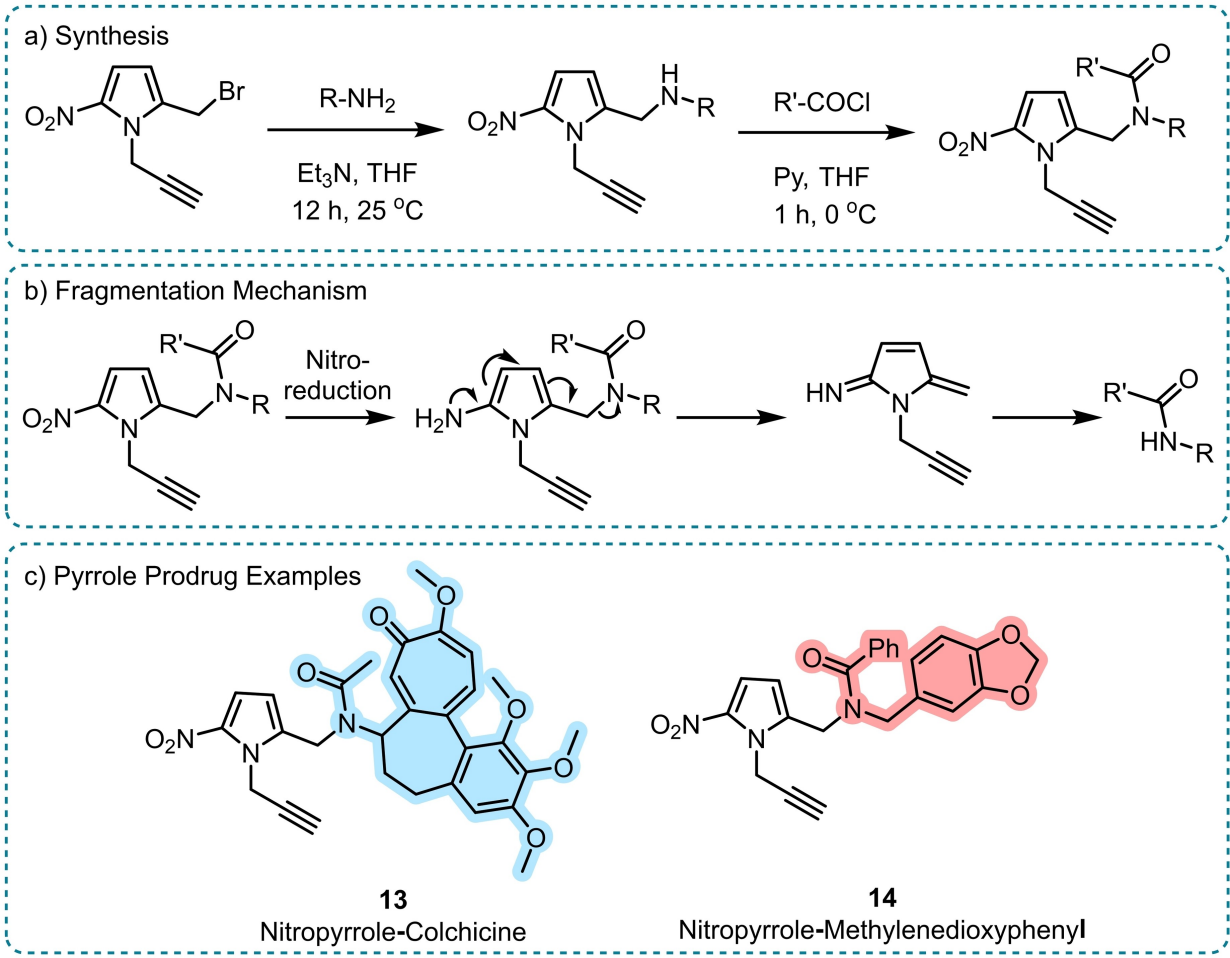
Amide release from nitropyrrole prodrugs.^[48]^ a) Synthesis, b) Reduction by Nitroreductase enzymes to an amine allows for 1,6‐elimination to release the amide, c) Example nitropyrrole‐colchicine prodrug **13** and methylenedioxyphenyl prodrug **14** which both exhibited full amide release within 2 hours of nitro reduction. The released drug is highlighted in each case. Py=Pyridine.

Most recently, Ermini *et al*. sought a method of amide release more akin to classical amine release using 1,6‐benzylic elimination by developing a nitropyrrole prodrug strategy (Scheme 8).^[48]^ It was noted that the classical 1,6‐benzylic elimination via anilines does not allow the spontaneous release of amides due their relatively high pKa (compared to carbamate R_2_NCOOH), therefore it was proposed that the use of heterocycles may enable amide release. Supported by computational analysis, it was found that aminopyrrole (accessed via nitro reduction from the corresponding nitropyrrole) was capable of releasing amides (Scheme 8b). The nitropyrrole trigger was also found to be compatible with the direct release of thiols and amines (classically released from disulphide bonds or carbamates, respectively), a challenging feat to achieve. The trigger system was exemplified on a range of secondary amides such as colchicine (**13**) and methylenedioxyphenyl (**14**) where full release could be shown within two hours of nitro reduction and good stability of these prodrugs in plasma and aqueous buffer was also observed (Scheme 8c).

## Summary and Outlook

In summary, whilst the number of strategies to release molecules via an amide motif is limited, the development of these methods has allowed for the creation of novel constructs with a variety of triggers and purpose. The key aspects of each method are summarised in Table 1.

**Table 1:**
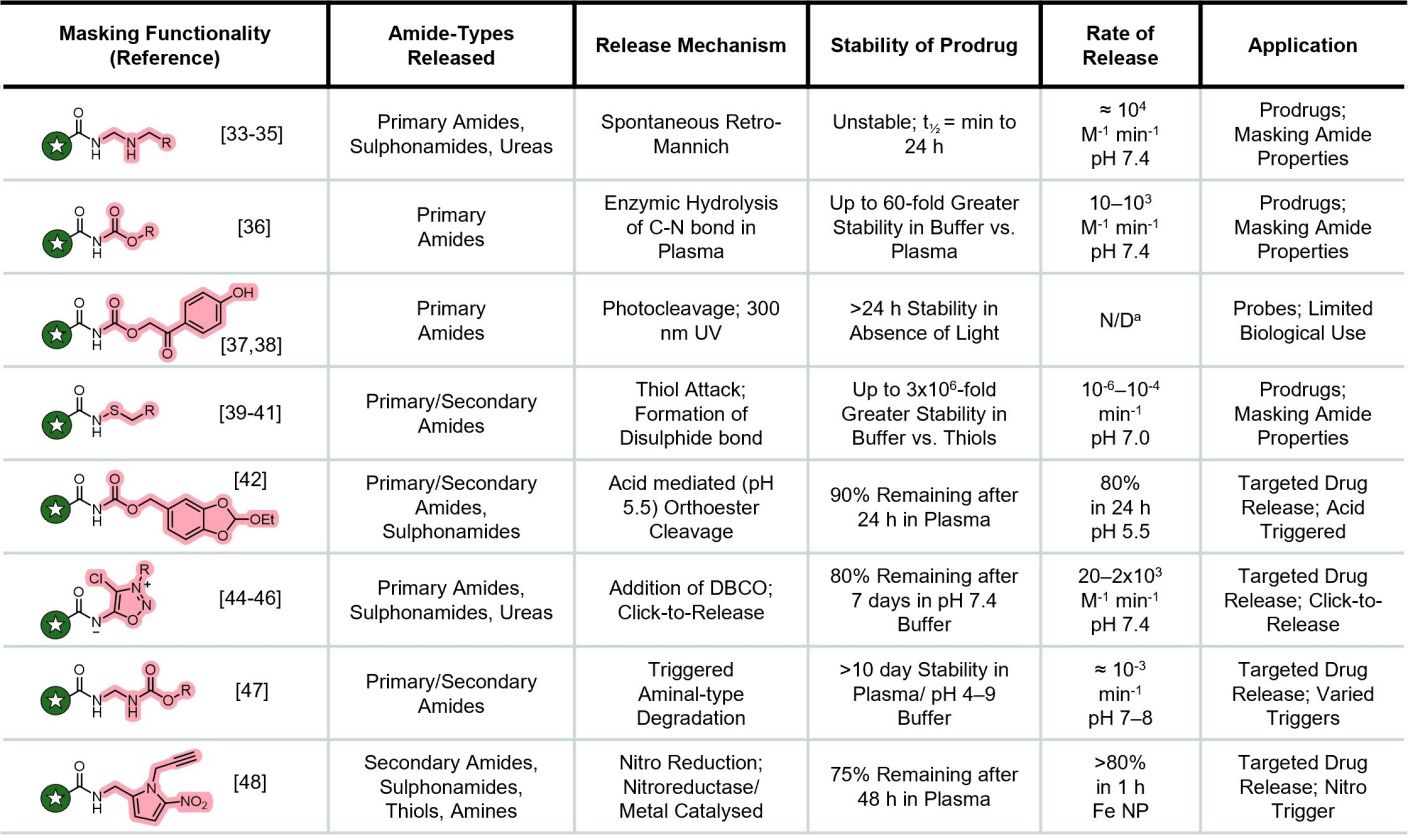
Overview of key aspects of the amide release methods described in this article. DBCO=Dibenzocyclooctyne, NP= Nanoparticles, N/D=Not Determined. a) No data reported for the p‐hydroxyphenacyl example.

Initial work in the field by Bundgaard and Stella focused on improving the properties of a parent amide by masking the moiety in a prodrug. This was achieved via the use of *N‐*aminomethyl and sulphenamide‐based capping groups to improve the solubility and permeability of the molecule. The capping groups were labile to hydrolysis or by thiol exchange, respectively, which allowed for the properties of the parent amide to be revealed. Whilst these strategies were shown to indeed improve the solubility of the parent drug, the instability of the prodrugs in physiological media precludes their wider use in amide prodrugs or ADCs.

Via the inclusion of bespoke triggers, Helquist, Petrini, and Feng successfully achieved the controlled release of a variety of amides facilitated by a greater stability of the linkages. Ermini *et. al* utilised a nitropyrrole trigger that was capable of releasing amide motifs via a classical 1,6‐elimination. Due to this mechanism the trigger also proved effective at directly releasing amines and thiols (usually released via carbamates and disulphide reduction, respectively), a generality not commonly reported. The aminomethyl carbamate linkage reported by Wharton *et al*. exhibited controlled amide release without the need for bespoke trigger motifs. Coupled with the stability of the linkage and scope of amide types released, this method displays the widest applicability of reported amide release strategies.

To the best of our knowledge, the strategies described in this concept article represent the entire field of amide release methods, clearly exemplifying the challenges in designing mask and linker motifs for amides. As with any self‐immolative system a considered choice of the trigger, linkage, and type of release must be made to ensure that the properties of the construct match favourably to its desired purpose. This could relate to the stability of the linkage, the rate of release, or the toxicity of any non‐payload fragments.

Amide‐release method development is in the early stages and so there is still research required to fully realise its potential. Compared to amine (via carbamate) and alcohol release, amide release rates are relatively slow and most methods require greater synthetic manipulation. This is mostly due to the different properties of amides compared to amines and alcohols but there is still room for improvement. The stability of the linkages in recently reported amide release methods, however, do compare well to the classical carbamate and ether based linkages for amine and alcohol release where excellent selectivity of payload release can be achieved. This compares favourably to many ester‐based alcohol release methods where premature hydrolysis is a common issue. In general, however, the true advantage of developing amide release methods is in expanding the range of functional groups that can be used as release handles, thus expanding the toolbox available for payload release. The future of the amide release field lies in three key areas:

Firstly, utilising amide release in the generation of effective prodrugs and ADCs. As exemplified by the release of linezolid, the amide represents the most attractive option for release not only due to the lack of other releasable structural motifs but also as the inclusion of extra bulk around the amide eliminates the drug's activity, thus fortuitously making the prodrug inactive. Further examples of amide release thus need to be explored and evaluated to realise the potential of the method.

Secondly, the rate of amide release should be increased. Many of the controlled amide release methods require around 24 hours to achieve extensive drug release, unfavourably comparing to amine release which can occur in less than 1 hour.^[27]^ Whilst a slower release may be beneficial in some cases for controlled drug dosing, issues may arise from the triggered prodrug being cleared or transported before the drug molecule has been cleaved, leading to off‐target effects.

Finally, the methods described should be adapted to allow for late‐stage functionalisation of the amide to add the trigger and linkage. Many of the methods described above require the cleavable linkage to be installed during the synthesis of the drug molecule. The ability to add the linkage onto the complete molecule would greatly expedite synthesis and testing of amide release, allowing libraries of amide‐containing molecules to be functionalised efficiently.

Overall, in the last few years great advancements in the release of amide containing molecules have been made with a plethora of bespoke and general triggers utilised to release a range of amide types. These systems have proved to have much greater selectively and stability than previous amide capping approaches and show great promise to allow the synthesis of novel and considered prodrugs, ADCs, and chemical probes.

## Conflict of Interests

The authors declare no conflict of interest.

1

## Biographical Information

*Thomas Wharton undertook his undergraduate study at the University of Leeds, graduating with a MChem which included a year in industry on the RiCH Internship program at Roche, Basel. Thomas carried out his PhD studies with Prof. David Spring at the University of Cambridge working on small molecule release systems and antibody‐drug conjugates. He is currently continuing this research as a postdoctoral research associate in the Spring group*.

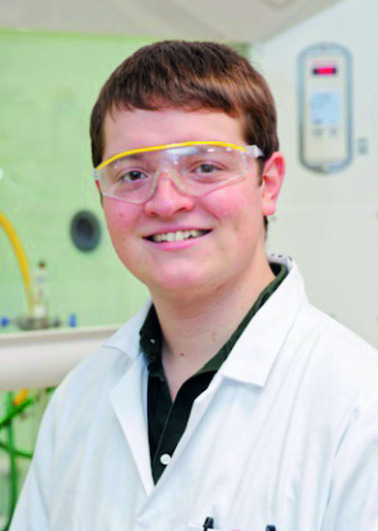

## Biographical Information

*David Spring is currently Professor of Chemistry and Chemical Biology at the University of Cambridge within the Chemistry Department. He received his DPhil (1998) at Oxford University under Sir Jack Baldwin. He then worked as a Wellcome Trust Postdoctoral Fellow at Harvard University with Stuart Schreiber (1999–2001), after which he joined the faculty at the University of Cambridge. His research programme is focused on the use of chemistry to explore biology*.

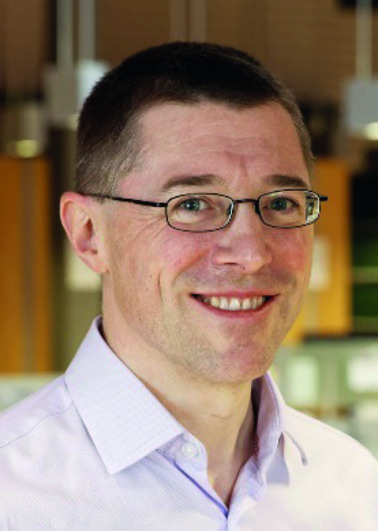

## Data Availability

Data sharing is not applicable to this article as no new data were created or analyzed in this study.
